# Combining Laue diffraction with Bragg coherent diffraction imaging at 34-ID-C

**DOI:** 10.1107/S1600577520009844

**Published:** 2020-08-11

**Authors:** Anastasios Pateras, Ross Harder, Wonsuk Cha, Jonathan G. Gigax, J. Kevin Baldwin, Jon Tischler, Ruqing Xu, Wenjun Liu, Mark J. Erdmann, Robert Kalt, Richard L. Sandberg, Saryu Fensin, Reeju Pokharel

**Affiliations:** a Materials Science and Technology Division, Los Alamos National Laboratory, PO Box 1663, Los Alamos, NM 87545, USA; b Advanced Photon Source, Argonne National Laboratory, Lemont, IL 60439, USA; c Center for Integrated Nanotechnologies, Los Alamos National Laboratory, PO Box 1663, Los Alamos, NM 87545, USA; d Department of Physics and Astronomy, Brigham Young University, Provo, UT 84602, USA

**Keywords:** Laue diffraction, Bragg diffraction, strain imaging, BCDI

## Abstract

Measurement modalities in Bragg coherent diffraction imaging (BCDI) rely on finding a signal from a single nanoscale crystal object which satisfies the Bragg condition among a large number of arbitrarily oriented nanocrystals. However, even when the signal from a single Bragg reflection with (*hkl*) Miller indices is found, the crystallographic axes on the retrieved three-dimensional (3D) image of the crystal remain unknown, and thus localizing in reciprocal space other Bragg reflections becomes time-consuming or requires good knowledge of the orientation of the crystal. Here, the commissioning of a movable double-bounce Si (111) monochromator at the 34-ID-C endstation of the Advanced Photon Source is reported, which aims at delivering multi-reflection BCDI as a standard tool in a single beamline instrument. The new instrument enables, through rapid switching from monochromatic to broadband (pink) beam, the use of Laue diffraction to determine crystal orientation. With a proper orientation matrix determined for the lattice, one can measure coherent diffraction patterns near multiple Bragg peaks, thus providing sufficient information to image the full strain tensor in 3D. The design, concept of operation, the developed procedures for indexing Laue patterns, and automated measuring of Bragg coherent diffraction data from multiple reflections of the same nanocrystal are discussed.

## Abstract 2

The commissioning of a movable X-ray monochromator at the 34-ID-C endstation of the Advanced Photon Source is reported. The new instrument allows indexing of nanocrystals with Laue diffraction and collecting multi-reflection BCDI data.

## 1. Introduction   

Macroscopic properties of crystalline materials depend on their atomic structure, dimensionality and other nanoscale phenomena. Dislocation locking, for example, is considered an effective physical mechanism to boost the hardness of polycrystalline metals (Spiecker *et al.*, 2019 ▸). Crystal slip has been shown to enhance surface diffusional creep and lead to unusual superplastic behavior of silver nanocrystals (Zhong *et al.*, 2017 ▸). Elastic constants are typically defined with respect to bulk while their values deviate and need to be measured explicitly in the case of two-dimensional materials or nanometre-sized objects (El-Atwani *et al.*, 2019 ▸; Gigax *et al.*, 2019 ▸). Particularly in micrometre- to nanometre-scale dimensions, the knowledge of the crystallographic orientation and full strain tensor are important pieces of information for predicting the mechanical properties of submicrometre particles and crystal grains of polycrystalline materials (Cherukara *et al.*, 2018*a* ▸). Bragg coherent X-ray diffraction imaging (BCDI) allows the visualization of the local atomic lattice displacement of single nanoparticles or grains in three dimensions. BCDI is compatible with operando measurements under different external stimuli, such as compression or tension, femtosecond laser light pulses, electric and magnetic fields allowing the visualization of evolving strain inside nanoparticles and ultimately the investigation of materials properties at the nanoscale (Clark *et al.*, 2013 ▸, 2014 ▸, 2015 ▸; Ulvestad *et al.*, 2015 ▸, 2017 ▸; Pateras *et al.*, 2019 ▸; Cherukara *et al.*, 2017 ▸; Björling *et al.*, 2019 ▸; Takahashi *et al.*, 2013 ▸; Newton *et al.*, 2019 ▸).

A frequent challenge faced in BCDI experiments is that it relies on satisfying the Bragg condition of a single-crystalline grain within a potentially large population of sub-micrometre scale crystals or grains with unknown crystallographic orientations. As a result, it is often extremely difficult to measure more than one Bragg peak, thus it is impossible to obtain all the components of the strain tensor (Newton *et al.*, 2009 ▸). For well faceted nanocrystals and when either the Miller indices of a measured reflection or the growth direction of the crystals is known in advance, it is shown that the orientation can be inferred by spinning the crystal until multiple reflections light up and indexing the pole figure using texture analysis algorithms (Richard *et al.*, 2018 ▸). Other strategies for collecting multi-reflection BCDI data include obtaining important information about the crystallographic orientation of an isolated specimen before a BCDI experiment from other techniques, either scanning extensive volumes of reciprocal space until a reflection is found or by performing Laue diffraction beforehand at a different instrument (Newton *et al.*, 2009 ▸; Hofmann *et al.*, 2017 ▸, 2020 ▸).

A broadband (pink) X-ray beam permits Laue diffraction patterns to be measured from a lattice. The pattern of reflections arising on a detector is then a direct fingerprint of the orientation of the crystal lattice, assuming one knows the structure of the unit cell of the crystal (Moffat *et al.*, 1984 ▸; Smallman & Ngan, 2014 ▸). In this way, one can determine the crystallographic orientations of arbitrarily oriented crystals (Warren, 2016 ▸; Larson *et al.*, 2002 ▸).

Here we present a new movable monochromator, recently commissioned at the APS 34-ID-C beamline, which allows a user to easily switch between a monochromatic and a broadband X-ray beam. We also present the developed procedures and capabilities that allow multi-reflection BCDI at a single beamline instrument. The concept of operation relies on obtaining the crystallographic orientations of arbitrarily oriented submicrometre crystals utilizing a broadband X-ray beam for Laue diffraction and a monochromatic beam for BCDI from different Bragg reflections (Cha *et al.*, 2016*a* ▸). The indexing of Laue patterns provides a detailed map of the crystal reflections in reciprocal space, assisting the localization of a desired Bragg peak. Then, by collecting at least three reflections, the three-dimensional (3D) image of the strain tensor of a nanocrystal can be obtained. This unique capability will be crucial for investigating properties of crystalline materials where the knowledge of the crystallographic orientation with respect to the axis of external stimuli is imperative (Pateras *et al.*, 2019 ▸; Newton *et al.*, 2009 ▸).

## 2. Design of the monochromator   

The 34-ID beamline at the Advanced Photon Source supports two experimental stations, a dedicated Laue diffraction microscopy instrument (34-ID-E) and the dedicated Coherent Diffraction Imaging instrument (34-ID-C). The 34-ID sector is fed by canted undulators with a 1 mrad angular separation between the beams. 34-ID-C, being the upstream instrument, has a shielded transport running through the station with a 300 mm separation between the beams. This separation is gained by both the cant of the sources and a flat, cryogenically cooled, platinum-coated mirror set at 5 mrad in the first optical enclosure of the beamline. The mirror filters the highest energies of the beamline’s 3.0 cm-period undulator providing X-rays up to roughly 17 keV at the endstation, with significantly lower intensity up to 25 keV. The total bandwidth of the incident beam can be increased by tapering the gap of the undulator magnets providing nearly 5 keV-wide harmonics. With the fundamental harmonic set to well below the energy cutoff of the mirror, Bragg peaks arising across the entire bandwidth of the undulator passed by the beamline optics can be observed in a single Laue pattern.

**Figure 1:**
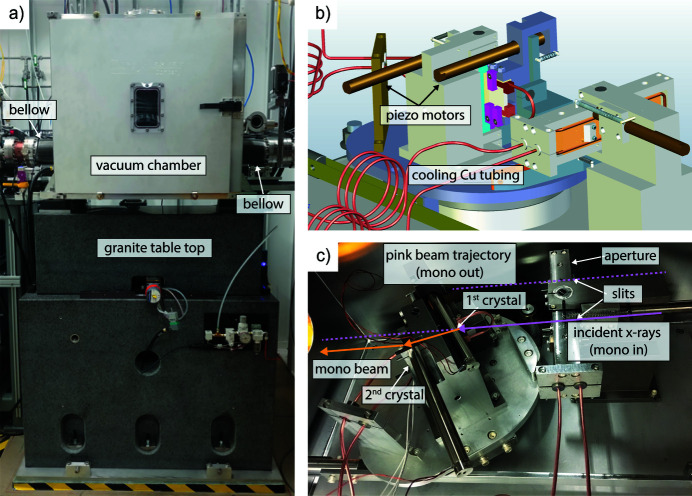
(*a*) Front view of the monochromator as it is placed at the 34-ID-C endstation. The vacuum chamber where the monochromator is enclosed sits on top of a granite stage. The granite table top is lifted by air bearings to permit a motor to slide the monochromator crystals in and out of the incident pink beam. The vacuum chamber is connected to the vacuum of the beamline with the use of two flexible stainless-steel bellows. (*b*) 3D rendered design of the two Si crystals with the piezo-actuated motors responsible for tilting the crystals. Also shown is the Cu tubing used for cooling the first crystal. (*c*) The monochromator crystals inside the vacuum chamber with the trajectory of the pink and monochromatic beam at the two monochromator positions. When the monochromator is in the trajectory of the incident X-rays (mono in) the monochromatic beam emerges from Bragg diffraction through the two horizontally scattering Si crystal wafers (Si crystals are vertical). When the monochromator is out (mono out) the pink beam passes undisturbed through the indicated slit.

A new small-offset (1 mm) double-bounce Si (111) monochromator was designed, installed and commissioned in the 34-ID-C endstation of the Advanced Photon Source. The movable monochromator is located inside a vacuum chamber on top of a granite stage to minimize vibrations and designed to operate within the 300 mm envelope afforded by the shielded transport to the downstream station. The new monochromator is located approximately 2 m away from the sample focus. A photograph of the entire assembly is shown in Fig. 1 ▸(*a*). Air bearings lift the granite table top to permit a motor to slide the monochromator crystals in and out of the incident polychromatic X-ray beam. The vacuum chamber is connected to the vacuum of the beamline with stainless steel bellows. By sliding the entire chamber perpendicular to the incident beam direction, the crystals are moved in and out of the pink beam, one can selectively switch from the broadband to the monochromatic beam produced by the Si crystals satisfying the Bragg condition for the energy of our choice. The monochromator can access X-ray energies from 6 to 20 keV. A detailed design rendering of the monochromator is shown in Fig. 1 ▸(*b*) and the as-built device depicting the trajectories of the pink and monochromatic beams is shown in Fig. 1 ▸(*c*). When the monochromator is in the trajectory of the incident X-rays (mono in) the monochromatic beam emerges from Bragg diffraction through the two horizontally scattering Si crystal wafers. When the monochromator is out (mono out) the pink beam passes through the indicated slit. The configuration is a standard horizontally reflecting artificial channel cut. The Si (111) oriented crystals are mounted upon a single rotation table. Precision alignment of the crystal planes is afforded by two precision piezo actuators, the first crystal having a fine rotation about the beam axis (chi) and the second crystal having a fine rotation about its Bragg angle (tweak).

The overall design goal was to have only piezo-actuated in-vacuum motions with all long-range, stepper-based motors mounted external to the vacuum chamber. This reduces both cost and the inevitable thermal drifts within the vacuum caused by power cycling of stepper motors. The Bragg angle of the monochromator is actuated by a linear stage mounted parallel to the X-ray direction driving a sine bar that is coupled to the crystal table through a stiff metal strap as seen in Fig. 1 ▸(*b*). The position of the sine bar is monitored with an optical encoder. The crystals are pre-aligned using fine-pitch screws adjusted against the pushers of the piezo devices.

The first crystal is clamped on the sides by copper water-cooling blocks and held in place by a three-point contact scheme far from the optically active surface. In addition, the second crystal of the monochromator is heated to increase the lattice constant of the silicon and modify the exit angle of the monochromatic beam, described in detail below. This is done to keep the monochromatic beam spatially aligned and coincident to the pink beam ensuring that the apparent source of both the monochromatic and pink beams appear in the same position in the final focusing optics of the instrument (Liu *et al.*, 2011 ▸). Therefore, one can switch from pink to monochromatic beam with confidence that the focused X-ray beam is on the same position of the sample. Beamline 34-ID-C uses bendable Kirkpatrick–Baez mirrors as the final focusing optic (Eng *et al.*, 1998 ▸). The typical spot size is 500 nm horizontal by 700 nm vertical on the center of rotation of the diffractometer, 7 cm downstream of the end of the horizontally focusing mirror.

In addition to the crystal carriage and rotation table, the monochromator contains an integrated beam-defining slit set. Both the monochromatic and pink beam-defining slits are mounted on a single carriage with a piezo actuator to pivot the slit blades and adjust the gaps, shown to the right in Figs. 1(*b*) and 1(*c*) ▸. The monochromatic slit was surveyed into position before the monochromator was installed in the beamline and aligned to the center of rotation of the crystal carriage. The slit gaps are designed for a range from 100 to 200 µm.

**Figure 2:**
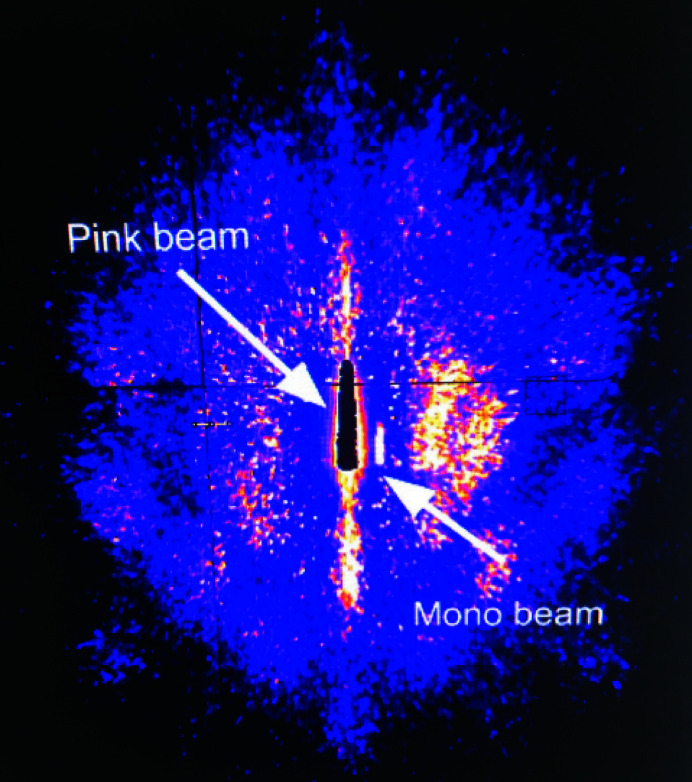
The direct pink beam passing through the first crystal of the monochromator and monochromatic beam as seen on the beamline GaAs sensor Timepix camera. The non-zero photon counts around the two beams originates from the scattering of a 1 mm-thick Pb foil used to attenuate the pink beam.

The initial commissioning image of both the pink and monochromatic beam on the beamline’s GaAs sensor Timepix camera is shown in Fig. 2 ▸. The pink beam appears black because the counters are being saturated by the high intensity, despite the pink beam being attenuated by the presence of the first crystal in its path. The measured separation between the beams is 16 pixels, corresponding to about 880 µm. There is an assumption that the symmetric reflections from the crystals do not alter the direction of the beam until we intentionally heat the second crystal as described in detail below. The first crystal also has a roll rotation adjustment. To set this we bring a horizontal edge into the beams and ensure the shadow of that edge appears at the same height in both beams on an image taken downstream. The image shown in Fig. 2 ▸ is the first image of th monochromatic beam from the device, once the second-crystal angle (tweak) was finally brought within the range of the piezo actuator. The energy of the undulator was 11 keV with a 2 mm taper, which led to a broader-bandwidth pink beam. With this configuration the required accuracy of the Bragg condition of the first crystal is significantly reduced.

To set the temperature of the second crystal a scintillation screen was placed at the center of the diffractometer and the position of the focused X-ray beams were directly observed with the beamline sample microscope. Since the X-ray focusing optics are left in place as the monochromator is inserted into the beam, the position of the focused beam at the center of the diffractometer will only shift if the apparent source position moves. Therefore, the pink and monochromatic beams must be entering the optic along the same axis if the focused spot does not shift when switching between pink and monochromatic beams. This circumstance is only possible if both the first crystal roll is adjusted properly and the second crystal is heated to shift the apparent position of the monochromatic source. After tuning, the required temperature of the second crystal is set to 85.00 ± 0.01°C using a polyimide heating film controlled by a Lakeshore 335 temperature controller. The temperature is stable to the second decimal digit.

## 3. Indexing of Laue patterns   

**Figure 3:**
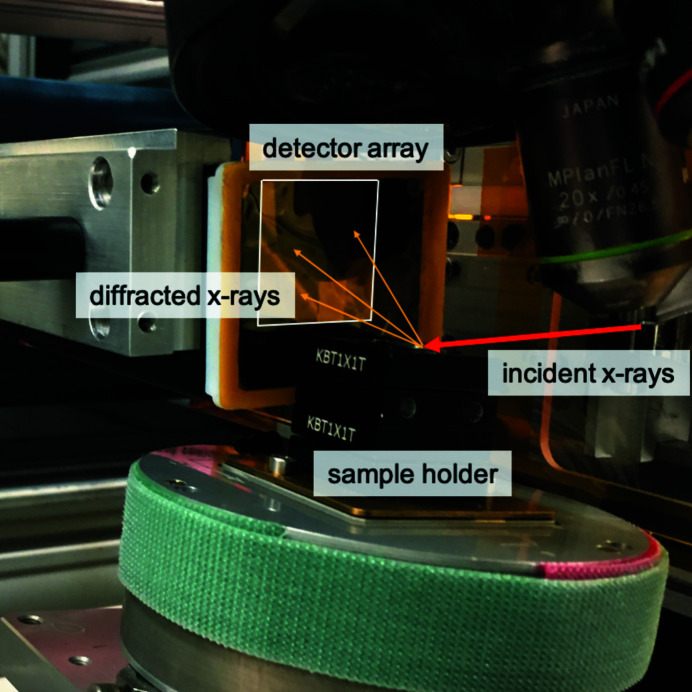
The experimental setup for collecting Laue patterns from Au nanocrystals at the 34-ID-C endstation of the Advanced Photon Source. The detector is placed 26 mm away from focus and vertically mounted. The sample is inclined to minimize the length of the beam footprint, and thus potential scattering from neighboring nanocrystals. The red arrow shows the pink beam, which is incident on the sample at 10° angle and the orange arrows depict the trajectories of the diffracted X-rays that contribute to the measured Bragg peaks of the Laue pattern.

To demonstrate the capability of indexing Laue patterns from arbitrarily oriented nanocrystals we used single-crystal submicrometre Au particles grown on Si (001) substrates. For the collection of Laue patterns, the experimental geometry depicted in Fig. 3 ▸ was used. An Amsterdam Scientific Instruments Timepix QTPX-262k detector with GaAs sensor was mounted vertically, 26 mm away from the center of rotation of the diffractometer as shown in Fig. 3 ▸. This detector is a single-photon-counting pixel array detector (512 × 512 pixels, 55 µm × 55 µm pixel size). Laue patterns were collected in the plane above and inboard of the incident beam (van Bakel *et al.*, 2013 ▸). The active area of the detector is 28.4 mm × 28.4 mm and at the given distance covers a solid angle of 57.3°. The detector is vertically mounted and the sample is inclined to the incident beam to minimize the length of the beam footprint, and thus potential scattering from neighboring nanocrystals.

**Figure 4:**
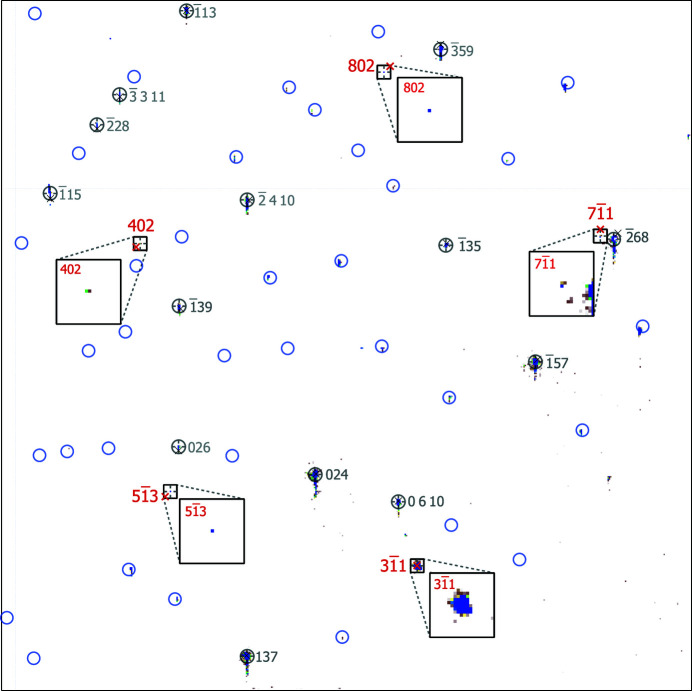
Indexed Laue pattern from a single-crystal Au nanoparticle on a Si substrate. The black squares are centered around the location of the Au peaks and the red labels correspond to the Miller indices of the indexed reflections. The red crosses indicate the position of the indexed reflection according to the fitting given by *LaueGo*. The indexed bright and vertically elongated peaks indicated by gray circles originate from the Si substrate and were considered in a separate list of peaks. The blue circles show the locations of the Si peaks which were not included in the list used for the indexing of the Laue pattern.

An example of an indexed Laue pattern is shown in Fig. 4 ▸. The Bragg reflections from the Si substrate are five orders brighter than the peaks from one Au nanoparticle. Our experiments showed that by repeatedly moving the sample 5 µm perpendicular to the beam, so the nanoparticle is in and out of the focused pink beam, we could observe the appearance and disappearance of the much dimmer and more difficult to observe corresponding set of reflections. Since the photon flux of the pink beam is roughly 30 times larger than that of the monochromatic beam, to collect the Laue patterns and visually identify the Au Bragg peaks we limited the number of acquisitions and exposure times down to 0.02–0.1 s. For total exposure times of less than 60 s, the illuminated crystals did not endure any visible radiation damage but occasionally we observed a limited number of nanocrystals, which rotated during exposure possibly due to radiation pressure (Kim *et al.*, 2016 ▸; Liang *et al.*, 2018 ▸). Particularly if compared with the several hours of exposure to the monochromatic beam for the collection of BCDI data, the effect of the pink beam was negligible.

The indexing of the identified Bragg peaks seen on the Laue pattern in Fig. 4 ▸ originating from the Au nanocrystal was performed using the *LaueGo* package (Larson *et al.*, 2002 ▸; Liu *et al.*, 2004 ▸, 2011 ▸). *LaueGo* uses a plane-search algorithm to identify the conics in the measured Laue pattern in reciprocal space (Busing & Levy, 1967 ▸; Ravelli *et al.*, 1996 ▸; Chung & Ice, 1999 ▸). In addition to the indexed Au Bragg peaks indicated by black squares with their Miller indices in red, we can also see the indexed Bragg peaks from the silicon substrate (gray circles). The indexed Si Bragg peaks consist of a list of peaks, which was used by *LaueGo* during the optimization. We also added gray circles around the indexed Si peaks and blue circles around the locations of Si peaks, which either are visible but have not been included in the list of peaks used for indexing or are missing because they do not light up due to the limited bandwidth of the pink beam. In the case of deliberately omitted peaks their locations coincide with the locations of the blue circles, verifying the result of the indexing.

The package takes as input the crystal structure space group of the material, the unit-cell dimensions and angles between the different crystallographic axes, the physical dimensions of the active area of the detector, the number of pixels, and the vectors **p** and **r**, which describe the position of the center of the detector with respect to the sample-beam interaction point for a specific experimental geometry. The vector **p** consists of the lateral offset of the center of the detector and the distance of the detector from the origin. The vector **r** is the Rodriguez rotation vector, which is applied on **p** to point at the coordinates of the center of the detector. While determining the values of **p** relies on accurately measuring the detector-to-sample distance down to a millimetre or less, determining the rotation vector **r** is done mathematically using the Rodriguez rotation formula (Koks, 2006 ▸). We determined the **p** vector to be **p** = [13.77, −0.55, −26.46] expressed in millimetres (mm), and the Rodrigues rotation vector **r** = [1.2, 1.2, 1.2] expressed in radians.

Providing the above information that accurately describes the experimental geometry and the crystal structure of Au (crystal space group, lattice constant 4.0782 Å) we were able to index the Laue pattern shown in Fig. 4 ▸ with root mean square error of 0.2°. The relatively large error can be further reduced, to the order of 10^−4^ with energy calibration. This part requires the precise determination of the energy of at least one of the experimentally observed reflections on the Laue pattern. However, for our purposes, 0.2° accuracy is sufficient since the coherent diffraction extends for nearly 1° about a given Bragg peak. The final result of the indexing contains a list of the indexed peaks with their Miller indices, energies, pixel coordinates on the detector, and the error with respect to the unstrained unit cell. The results are summarized in Table 1 ▸.

**Figure 5:**
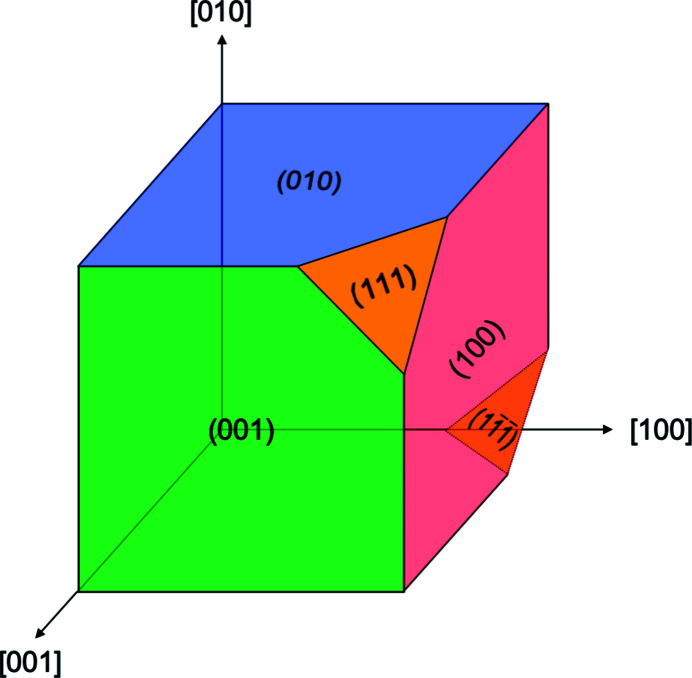
A schematic depicting the crystallographic directions in square brackets and families of atomic planes in parentheses of a simple cubic crystal system.

The most important information obtained by the indexing of a Laue pattern is the orientation matrix U_c_. The orientation matrix obtained from the Laue pattern of Fig. 4 ▸ contains the reciprocal space lattice vectors 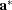, 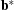, 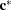 in the crystal frame given in units of inverse nanometres (nm^−1^),
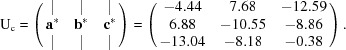
While the orientation matrix U_c_ contains the necessary information of how the crystal is oriented in reciprocal space, it does not consist of a convenient representation in order to easily navigate from one Bragg reflection to another. Thus, we will translate this information to the real space coordinate system of the laboratory, and, more specifically, to a set of two real-space vectors, one with the out-of-plane direction of the scattering planes and one with one in-plane direction, denoted by 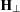 and 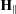, respectively. To translate the orientation matrix U_c_ into the two vectors, we first apply the relative rotation matrices R_θ_, R_ϕ_ and R_χ_ based on the values of the angles θ, ϕ and χ of the goniometer. This gives us a new matrix expressed in the laboratory frame,
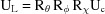
with
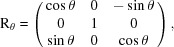


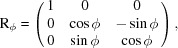


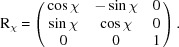
The 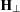, 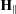 vectors are obtained if we multiply the unitary vectors, and in the laboratory frame with the transpose of the U_L_ matrix (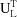),
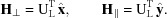
The calculation gives us for the experimentally determined orientation matrix, U_c_, the following vectors,
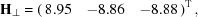
which means that the scattering planes are as illustrated in Fig. 5 ▸, and
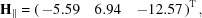
nearly equivalent to a crystallographic direction.

The 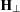, 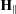 vectors are then inserted into *Spec* as known orientations, enabling the angle calculation of any desired Bragg reflection (Hofmann *et al.*, 2017 ▸). Fig. 5 ▸ illustrates different sets of atomic planes for the given orientation of the unit cell, which can contribute to intensity patterns in specific directions of 3D space. By possessing the out-of-plane vector of the diffracting planes, 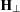, and one in-plane vector 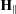, *Spec* has all the necessary information for locating other Bragg peaks, as long as they are within the permitted angular range of motion of the sample and detector goniometers. For example, by knowing that the family of planes is contributing to scattering, we can rotate our crystal as needed in θ, ϕ and χ in order to measure a (001) reflection (Hofmann *et al.*, 2017 ▸).

## 4. Calculation of the strain tensor   

**Figure 6:**
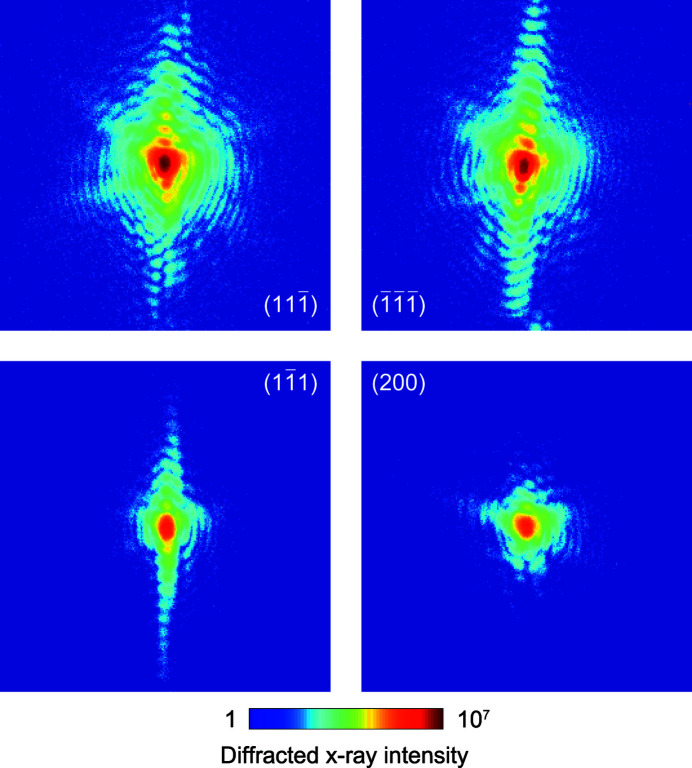
Measured X-ray diffraction patterns from a single Au nanoparticle for four different Bragg reflections. The patterns were taken at the intensity maximum of the rocking curve during the BCDI measurement.

**Figure 7:**
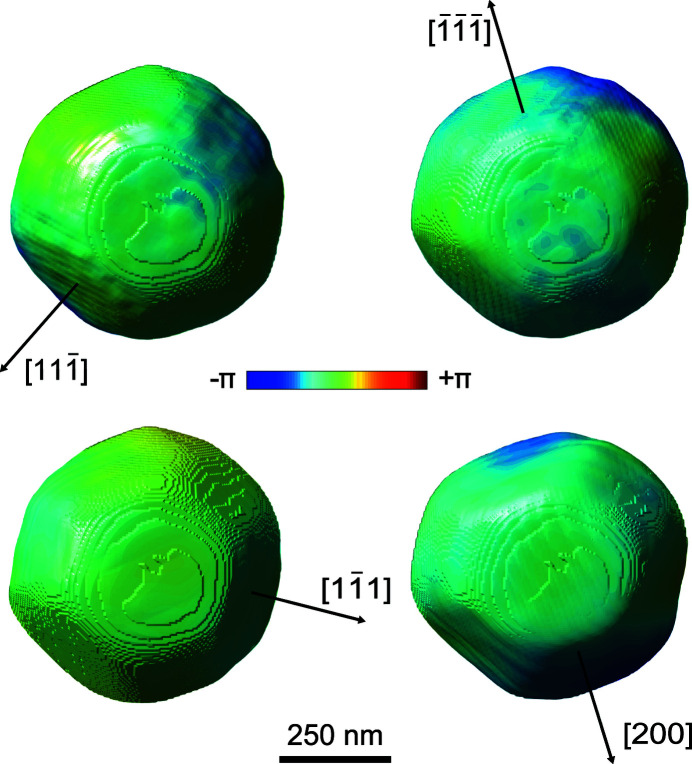
Three-dimensional reconstructions of the measured Au nanocrystal from four different Bragg reflections.

Using the indexing result from the Laue pattern of Fig. 4 ▸ we found four Bragg reflections originating from the same single-crystal Au nanoparticle, simply by typing the desired Miller indices. Fig. 6 ▸ shows slices through the measured 3D X-ray diffraction patterns at the maximum intensity for the 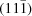, 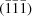, 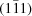 and (200) Bragg reflections obtained with the monochromatic beam at 9 keV. The patterns demonstrate high-visibility fringes that extend far in reciprocal space from the sharp facets of the Au particle, features that assist obtaining high-resolution BCDI reconstructions (Cherukara *et al.*, 2018*b* ▸). The lower intensities of the 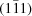 and (200) reflections observed in Fig. 6 ▸ are due to accidentally operating the undulator in tapered mode. This led to a broader distribution of photons over the energy spectrum, and thus an order of magnitude decrease in the diffracted intensity observed on the detector screen, which originates from the narrow energy bandwidth that satisfies the Bragg condition for our single-crystal nanoparticle. We considered the observed intensity fluctuations in the strain tensor reconstruction algorithm, which were found to have a negligible effect on the final reconstructed phases. For all the collected datasets from the four Bragg reflections, we reconstructed the 3D crystal electron density shown in Fig. 7 ▸. The shape, dimensions and surface features observed at 20% of maximum amplitude isosurface of the Au nanocrystal are consistent among the reconstructions from the different reflections.

In addition to the 3D crystal electron density, we plot the arrows indicating the crystallographic orientation axes for each measurement. The arrow is normal to the (*hkl*) family of atomic planes, which contributes to the scattering signal. A series of alternating error-reduction and HIO algorithms was used for 620 iterations (Clark *et al.*, 2015 ▸) and the phase-retrieval transfer function was employed for the calculation of the resolution of the obtained reconstruction (Ulvestad *et al.*, 2015 ▸; Chapman *et al.*, 2006 ▸). We estimated an isotropic resolution for all three dimensions of approximately 15 nm, slightly larger than the voxel size of 11 nm. Comparison of the reconstructed shapes was made with the computational methods previously developed by Hofmann *et al.* (2017 ▸).

**Figure 8:**
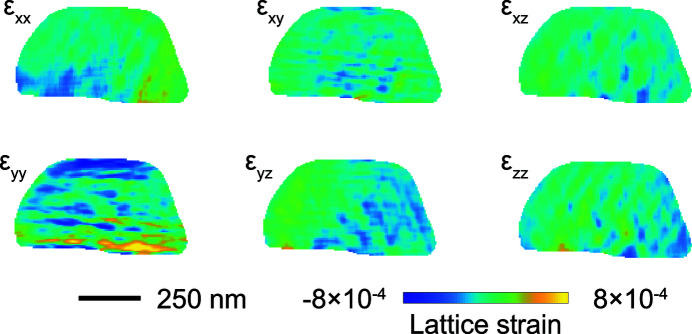
Two-dimensional slices of the strain components taken at the center of the reconstructed volumes of the Au nanoparticle.

Having all components of the crystal atomic displacement we can calculate the entire Eulerian strain tensor ∊_*ij*_, given by the following relation (Newton *et al.*, 2009 ▸),
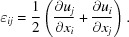
The result of the calculation is plotted in Fig. 8 ▸, where slices are taken at the center of each of the 3D reconstructions. Each slice depicts the different crystal strain components ∊_*ij*_ of the strain tensor (Hofmann *et al.*, 2020 ▸). The reconstructed slices show in general the presence of small strain fields inside the crystal on the order of 10^−4^, except in the case of the ∊_*yy*_ component, for which the bottom part of the Au particle reaches values up to 8 × 10^−4^. This is likely due to stress transmitting through the interface of the Au particle with the silicon substrate (Pfeifer *et al.*, 2006 ▸; Pateras *et al.*, 2018 ▸). In comparison with other optical methods for measuring full-field displacements and strains, such as digital image correlation, BCDI provides strain information in 3D allowing the investigation of defects or other nanoscale phenomena inside a single grain (Miao *et al.*, 2015 ▸; Hung Lo *et al.*, 2018 ▸).

## 5. Conclusions   

The commissioning of a movable X-ray monochromator at the 34-ID-C endstation of the Advanced Photon Source will change dramatically the user workflow, from sample preparation to measurement strategies, data analysis and time management during the beam time. Initially it will require modifying the design of samples since particularly dense specimens make the indexing of Laue patterns a challenging problem. This is due to the extents of high-intensity tails around the focused beam caused by poor surface quality of the focusing mirrors. Crystals that are several micrometres away from the focal spot can produce Laue patterns that are visible on the detector. However, strategies for identifying Laue patterns from multiple grains within the beam are being developed. Future potentially beneficial developments could include integrating machine learning algorithms to assist indexing of complex Laue patterns (Zhou *et al.*, 2018 ▸) or 3D reconstructions based on variable-wavelength coherent X-rays rather than on rocking crystals (Cha *et al.*, 2016*b* ▸; Lauraux *et al.*, 2020 ▸). These developments could improve standardization of each experiment, minimize the man-hours spent finding signals for experiments, and facilitate faster analysis and interpretation of numerous datasets. In addition, we continue to streamline the workflow to orient samples and measure coherent diffraction. We intend to further integrate *LaueGo* directly into the beamline controls system, making the orientation step of arbitrary samples seamless to the user.

## Figures and Tables

**Table 1:** Miller indices of the indexed reflections from the Au nanoparticle, their energies and error

(*hkl*)	Energy (keV)	Error (°)
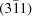	8.21	0.07
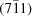	14.28	0.16
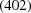	8.86	0.14
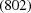	15.14	0.48
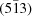	14.10	0.39
